# Factors Affecting Family Presence During Fracture Reduction in the Pediatric Emergency Department

**DOI:** 10.5811/westjem.2018.9.38379

**Published:** 2018-10-18

**Authors:** Albert Zhang, Regina M. Yocum, Michael D. Repplinger, Aimee T. Broman, Michael K. Kim

**Affiliations:** * University of Minnesota, Department of Pediatrics, Minneapolis, Minnesota; † American Family Children’s Hospital, Department of Child Life Services, Madison, Wisconsin; ‡ University of Wisconsin, Madison, BerbeeWalsh Department of Emergency Medicine, Madison, Wisconsin; § University of Wisconsin, Madison, Department of Radiology, Madison, Wisconsin; ¶ University of Wisconsin, Madison, Department of Pediatrics, Madison, Wisconsin; || University of Wisconsin, Madison, Department of Biostatistics and Medical Informatics, Madison, Wisconsin

## Abstract

**Introduction:**

Asking family members to leave during invasive procedures has historically been common practice; however, evidence-based recommendations have altered the trend of family presence during pediatric procedures. The aim of this study was to determine factors related to family members’ choice to be present or absent during fracture reductions in a pediatric emergency department (ED), and their satisfaction with that choice.

**Methods:**

We administered role-specific, anonymous surveys to a convenience sample of patients’ family members in the ED of a Level I pediatric trauma center. All family members were given a choice of where to be during the procedure.

**Results:**

Twenty-five family members of 18 patients completed surveys. Seventeen family members chose to stay in the room. Family member satisfaction with their decision to be inside or outside the room during the procedure (median = very satisfied) was almost uniformly high and not associated with any of the following variables: previous presence during a medical procedure; provider-reported procedure difficulty, or anxiety levels. Family member perception of procedure success (median = extremely well) was also high and not associated with other variables. Location during the procedure was associated with a desire to be in the same location in the future (Fisher’s exact test, p=0.001). Common themes found among family members’ reasons for their location decisions and satisfaction levels were a desire to support the patient, high staff competence, and their right as parents to choose their location.

**Conclusion:**

Family members self-select their location during their child’s fracture reduction to high levels of satisfaction, and they considered the ability to choose their location as important.

## INTRODUCTION

Patient- and family-centered care (PFCC) refers to “health care that is compassionate, includes patients and families as partners and collaborators, is provided with respect, and treats patients and families with dignity.”^1^ The Institute of Medicine states that patient-centered care is geared toward “providing care that is respectful of and responsive to individual patient preferences, needs, and values, and ensuring that patient values guide all clinical decisions.”^2^ Two important aspects of PFCC are family education and presence during patient care and treatment.

Although asking family members to leave during invasive procedures has historically been common practice, evidence-based recommendations have altered the trend of family presence during pediatric procedures. For instance, studies have refuted the misperceptions that family members may interfere during the procedure, that the procedure may cause great distress to them, or that they do not have a preference regarding their own presence.^3^–^7^ Other studies have suggested that a provider’s preference against family member presence is correlated with that provider’s lack of experience having family present and that providers’ views on family presence differ from patients’ views.^8^–^14^ In fact, family presence may have beneficial effects on the patient-doctor relationship and patients’ medical outcomes.^15^,^16^

Despite these findings, few studies have investigated family member presence during fracture reductions and other orthopedic procedures, which are common in emergency departments (ED). Orthopedic procedures are unique among procedures as they are commonly performed in the ED, and frequently require procedural sedation and analgesia. However, the graphic nature of the procedure is often considered a reason to exclude family presence. PFCC, because it calls for collaboration with patients’ families as partners, demands a challenge to the assumption that orthopedic procedures are difficult for family members to tolerate and may cause undue distress. Although there is literature assessing providers’ views on family presence during fracture reduction, there is a gap in knowledge regarding factors affecting family members’ preferences and decisions regarding whether to be present during fracture reduction.^17^ To that end, this study aimed to identify factors that affect the decision to stay in a patient’s room during a fracture reduction as well as to describe family members’ self-reported experience during the procedure.

## METHODS

### Study Design and Setting

This study was a prospective, observational survey study of a convenience sample of family members and providers of pediatric patients (i.e. less than 18 years old) undergoing fracture reductions in a tertiary-care pediatric ED with an average of 12,000 visits annually. After identification by ED providers, eligible family members were approached for enrollment based on the availability of the research assistant. The research assistant was a medical student on a summer research elective and was scheduled to be available 40 hours per week, during typical “daytime hours” (i.e., 9 a.m.–5 p.m.). Our institution has implemented many PFCC guidelines, a component of which recommends allowing pediatric patients’ family members to choose whether to be inside or outside the procedure room before, during, and after fracture reductions.

**Population Health Research Capsule**

What do we already know about this issue?

Providers overwhelmingly accept family presence during many emergency procedures, though this is less common during fracture reductions.

What was the research question?

To characterize location preference and experience of family members during pediatric fracture reductions.

What was the major finding of the study?

Family members self-selected their location with high satisfaction and stressed the importance of the choice.

How does this improve population health?

Delivering effective patient- and family-centered care, and building mutual trust and increased satisfaction require understanding of families’ preferences and values.

### Survey Design and Development

Survey instruments (one for pre-procedure, one for post-procedure) were created in consultation with the survey center affiliated with the study’s parent university (Appendix). Family members were asked about factors that could contribute to their choice to be present or absent during fracture reductions in a pediatric ED and their satisfaction with that choice. These factors included relationship to patient, previous presence during a medical procedure, preference for being inside or outside the room during the procedure, and anticipated anxiety level during the procedure. Actual location (i.e., inside or outside the room) during the procedure was also recorded for comparison. The post-procedure survey assessed the actual level of anxiety felt during the procedure, impression of how well the procedure went, and location preference for future fracture reductions (i.e., inside or outside the procedure room).

Of note, after reviewing the results of the first 10 surveys, we modified the post-reduction survey to better assess family members’ satisfaction with the procedure. We replaced, “Where would you recommend parents/family members of other children to be during the same procedure?” and “Do you want to be given the option to be in or outside the procedure room for all procedures performed on family members?” with the following: “How satisfied are you with the way the staff prepared you for the procedure?”; “How satisfied are you with your choice to be inside/outside of the procedure room during the procedure?”; and “How important is the option to be in or outside the room for all procedures performed on family members?”

### Survey Administration

The principal investigator administered paper surveys during the research work hours before (pre-reduction survey) and after (post-reduction survey) the procedure to eligible family members accompanying pediatric patients undergoing fracture reduction. Survey participants were informed of the purpose. No protected health information was gathered in this study.

### Data Analysis

This is a descriptive study in which we display the association of pre-procedure factors compared with patients’ family members’ preferences for being inside or outside the procedure room during fracture reductions. We also observed the influence of actual location (inside or outside the procedure room) compared to post-procedure measures of satisfaction, such as overall impression of how well the procedure went, anxiety during the procedure, and location preference for future procedures. Furthermore, we examined family members’ future location preferences when considering their perceived anxiety, actual location during the procedure, and initial location preferences. Finally, we asked family members the level of importance that they placed on having the choice to be present during the procedure. Quantitative data are reported as raw percentages and we used Fisher’s exact test to determine the strength of association (though this should be viewed as exploratory only since we did not power this study to establish causation). As mentioned previously, due to the small number of patients and family members, all analyses should be considered descriptive.

For qualitative analysis, we performed conventional content analysis on responses to qualitative questions. This involved first reviewing answers to free-response questions and then creating de novo response categories based on common thematic elements among responses to the same question.

## RESULTS

### Characteristics of Participants

There were 25 family members accompanying 18 patients who completed surveys. Patient age ranged from 4–16 years old, with median age of nine (Table 1). A majority of the fractures were in either forearm (n=13, 72%), and a majority of patients were administered ketamine for sedation (n=16, 89%). Fourteen (78%) patients had at least one family member who stayed in the room during the procedure.

Twenty-one (84%) of the 25 family members completed pre-procedure surveys and all 25 (100%) completed post-procedure surveys. Of the 18 reductions performed, a child life specialist was present during the procedure for 14 (77.8%). As mentioned in the methods section, the post-reduction survey was modified part way through the study and 12 of 25 family members completed this revised post-survey.

### Main Results

There was no statistical difference between family member type (e.g., mother, father, other) and their actual location during the procedure: Mothers remained in the room in 86% of cases compared to 50% for fathers (p=0.08). There were four family members with missing values for location preference before the procedure; if we assume that their location preference was honored, almost everyone’s preference was honored (80% of those who preferred to be inside ahead of the procedure stayed inside, and 100% of those who wished to remain outside the procedure room did [Table 2]). We observed no strong relationship of anticipated anxiety to choice of location, although those who anticipated lower anxiety were observed to be more likely to remain inside the procedure room (80% vs. 55.6%, p=0.35). Location during the procedure did not affect the family member’s impression of how well it went; everyone who responded said it went “very well” to “extremely well” (Table 3). A majority of those who stayed inside during the procedure described having lower anxiety than those who were outside the room (Table 4).

When asked where they would like to be during a similar procedure in the future, everyone who stayed inside said they would choose to do so again; about half of those who stayed outside said they would do so again (Table 5) (p=0.001). Regarding the importance of the option to choose to be inside or outside of the procedure room, four of the 12 who responded (33%) thought the option was “extremely important,” seven (58%) thought it was “very important,” and one (8%) felt it was “somewhat important.”

### Qualitative Outcome Measures

Regarding their satisfaction with their location during the procedure, 10 of the 12 family members responded that they were “very satisfied” (83%) while one was “somewhat satisfied” (8%) and another “neutral” (8%). The “somewhat satisfied” response came from a mother who remained inside the procedure room and stated, “It was hard to watch but still glad we were in the room.” She also added that the “doctor and nurses made sure she [her daughter] was very comfortable. They also took their time making sure arm was perfectly back aligned.” The mother wanted to be inside the procedure room in the future, writing that “being there was reassuring knowing she [her daughter] was ok.”

The “neutral” response came from a mother who was outside the room during the procedure and had no preference as to her location in the future. She wrote: “He [her son] did fine without me. I was glad to not be exposed to the radiation.” She marked “not at all” for her actual anxiety level and thought the procedure went “very well,” noting “no pain, kind staff, accommodating my need to get food for patient.”

Several themes emerged from family members’ explanations of their experience. The most common reason for parents deciding to stay in the procedure room was to “be there” for their child. Of the 18 family members who reported wanting to be inside the procedure room on their pre-procedure survey, 15 (83.3%) cited a desire to be present as a support to their child. One respondent wrote that she wanted “to be there for my child so she feels comfortable and loved.” Another wrote that it was “easier to be with child than away worrying.”

When asked to justify the importance of having the choice to be inside or outside of the procedure room for any procedure, 11 of 12 family members felt that it was very or extremely important and emphasized the benefits of having a choice. One respondent wrote, “Every parent has the right/responsibility to be there for support and protection.” Another wrote, “Allowing family to be witness and in the room allows for a resemblance of control. Kicking the parents out only makes them worry more.” Twenty-five percent of all family members also cited personal preference toward having the choice: “good to have a choice;” “I am glad I had the option;” and “some people would prefer to be with their child.”

## DISCUSSION

In this descriptive study of family member presence during pediatric fracture reductions in the ED, we found that family members largely 1) prefer to be inside the room during the procedure; 2) prefer to be in the same location for future procedures; and 3) believe it is important to be asked where they would prefer to be during the procedure. Studies on family presence during fracture reductions in the pediatric ED are limited. Most available literature focuses on family presence during pediatric resuscitation or other more invasive procedures.^14^,^18^,^19^ Our study begins to address the need for procedure-specific studies focusing on the experience of family members, particularly as it relates to having a choice of location during procedures.

In our study, family members self-selected their location to a high level of satisfaction, regardless of what that choice was. Not only was satisfaction with location almost uniformly high among family members, but location during the procedure was also highly associated with the desire to be in the same location in the future. Understanding and accommodating this strong association may be an important factor in the development of PFCC guidelines in the ED.

Our results differ somewhat from Gamell et al., who conducted a survey study in an ED in Barcelona, Spain. Of their respondents, 86.5% expressed a desire to stay during fracture reduction, while only 37% actually stayed. Also, only 51.6% of parents believed that they should have the choice to be present.^20^ This discrepancy in responses, particularly between the desire to stay and to have the choice to be present, may be attributable to many factors, including differences in culture, facility resources and institutional guidelines, but warrants further investigation into reasons behind each desire and how those desires might be reconciled.

Our results suggest that family members’ positive impressions of procedure success were independent of family member location during the procedure; instead, positive impressions of success were associated with perceived staff competence. Responses from family members who stayed with the patient suggest that being inside the room enhanced family members’ positive impressions. This likely informed their high levels of satisfaction. Regardless of the location, family members emphasized the importance of effective communication from staff regarding procedure progress and procedure success. This supports various studies that demonstrated effective provider communication shapes and improves family member and pediatric patient experience.^21^–^23^

There were four family members whose future location preference differed from the actual location. Of these four cases, it appeared that staying in the room was uncomfortable for them and they chose to leave, but indicated they still would like to be inside the room in the future. If family members find the procedure more distressing than expected, thorough pre-procedure education should inform them that they could ask for help or choose to step out at any time. This in turn will lead to self-monitoring of family members to inform staff if they need to leave the room.

In addition to a family presence guideline for fracture reduction, we routinely allow parents to remain with patients when radiography is performed, though parents are required to wear a lead apron if they remain in the room. Unfortunately, we did not ask about family members’ concerns regarding exposure to ionizing radiation in this study. We also did not consider the presence of multiple family members since our guideline recommends only one family member to be in the room during procedures. Although not being present during the procedure may lead to lower satisfaction, knowing that at least one family member is present may be a source of reassurance for any others accompanying pediatric patients. We also did not consider socioeconomic and ethnic perspectives of patients and families in our study. All of these factors require additional consideration in future studies.

Based on the recommendations from the American College of Emergency Physicians and American Academy of Pediatrics, which support PFCC, and our own institutional experience, we feel that it is important to invite family member presence during pediatric fracture reductions. Guidelines regarding PFCC as it relates to procedures in the ED should consider family member preference and resource availability (e.g., child life specialists) in their recommendations. They should also strongly support communication between family members and care providers.

## LIMITATIONS

This observational, descriptive study had several limitations. First, there was the potential for selection bias arising from convenience sampling. Second, our survey instruments were not previously validated, raising concern for possible information bias, although we constructed them with the help of methodological experts. Third, our study took place in a tertiary-care, pediatric ED with ample resources such as child life specialists, which may limit generalizability. Fourth, family members’ answers to our survey may have been subject to information bias if they did not want to admit they had made the “wrong choice” for themselves. Lastly, the change in the post-reduction survey mid-recruitment resulted in fewer responses to some of the questions, but we felt that the modified questions provided more insight into family members’ satisfaction with the procedure.

## CONCLUSION

In our study we did not find any factors associated with family preference to be present during fracture reduction in children. However, it was very important to family members to be given the option to be present with the child. Regardless of their pre-procedure location preference and actual location during the procedure, they uniformly experienced high levels of satisfaction.

## Supplementary Information



## Figures and Tables

**Table 1:** Patient characteristics (n=18) in survey of factors affecting family presence during procedure.

Patient characteristics	Number of patients (%)
Age	
4–5	4 (22.2%)
6–10	6 (33.3%)
11–15	7 (38.9%)
16	1 (5.6%)
Sex	
Female	7 (38.9%)
Male	11 (61.1%)
Fracture type	
Both forearm bones	13 (72.2%)
Distal radius	1 (5.6%)
Ankle	3 (16.7%)
Finger	1 (5.6%)
Anesthesia type	
Regional anesthesia	2 (11.1%)
Sedated with ketamine	16 (88.9%)
Number of family members in room during procedure	
0	4 (22.2%)
1	11 (61.1%)
2	3 (16.7%)

**Table 2:** Status of patient’s family member before procedure compared with location during procedure.

Family member characteristics	Inside	Outside	Fisher’s p value
Relationship			
Mother	12 (85.7%)	2 (14.3%)	0.083
Father	4 (50%)	4 (50%)	0.083
Other	1 (33.3%)	2 (66.7%)	0.083
Location preference before procedure			
Inside	16 (80%)	4 (20%)	0.0055
Outside	0 (0%)	4 (100%)	0.0055
No preference	1 (100%)	0 (0%)	0.0055
Anticipated anxiety before procedure			
0–1 “None” to “a little”	8 (80%)	2 (20%)	0.35
2–4 “Somewhat” to “a great deal”	5 (55.6%)	4 (44.4%)	0.35

**Table 3:** Family member’s location during procedure compared with impression of how well the procedure went.

Location	Very well	Extremely well
Inside	7 (41.2%)	10 (58.8%)
Outside	4 (50%)	4 (50%)

**Table 4:** Family member’s location during procedure compared with anxiety level reported.

Location	“None” to “a little” (0–1)	“Somewhat” to “a great deal” (2–4)
Inside	10 (58.8%)	7 (41.2%)
Outside	2 (25%)	6 (75%)

Fisher’s exact test for count data, p=1.

Fisher’s exact test for count data, p=0.2016.

**Table 5:** Family member’s location during procedure compared to reported future location preference.

Location	Inside	Outside	No preference
Inside	17 (100%)	0 (0%)	0 (0%)
Outside	3 (37.5%)	4 (50%)	1 (12.5%)

Fisher’s exact test for count data, p=0.001.
