# Supplementary material for: Factors Affecting Family Presence During Fracture Reduction in the Pediatric Emergency Department
Source: West J Emerg Med. 2018 Oct 18;19(6):970–6. doi: 10.5811/westjem.2018.9.38379 (PMC6225943; doi:10.5811/westjem.2018.9.38379)
Supplement: Supplementary file 1 [file wjem-19-970-s001.docx]

**APPENDIX: Survey Instruments**

**Pre-Procedure Survey for Family**

**Q1 How are you related to the patient that will undergo today’s procedure?**

- Mother
- Father
- Sibling
- Friend
- Other (please specify):

**Q2 Have you ever stayed with a child or family member through a medical procedure?**

- Yes
- No

If you answered “Yes” to Q2, please respond to Q3-Q5.

If you answered “No” to Q2, you may ignore Q3-Q5 and proceed to Q6.

**Q3 If yes, what procedure?**

**Q4 Was your child or family member given medication to be calm or sleepy?**

- Yes
- No

**Q5 How old was your child or family member?**

**Q6 Has today's procedure been fully explained to you?**

- Yes
- No

**Q7 Where would you prefer to be during the procedure?**

- In the procedure room
- Outside of the procedure room
- No preference
- Unsure

**Q8 What is the reason for your answer to Q7?**

**Q9 During the procedure, how anxious do you expect to feel?**

- Not at all
- A little
- Somewhat
- Quite a bit
- A great deal

**Q10 What is the reason for your answer to Q9?**

**Q11 Do you have any additional comments or concerns?**

**Post-Procedure Survey for Family**

**Q1 How are you related to the patient that underwent today's procedure?**

- Mother
- Father
- Sibling
- Friend
- Other (please specify):

**Q2 What is your impression of how well the procedure went?**

- Not well at all
- A little well
- Somewhat well
- Very well
- Extremely well

**Q3 What is the reason for your answer to Q2?**

**Q4 During the procedure, how anxious did you feel?**

- Not at all
- A little
- Somewhat
- Quite a bit
- A great deal

**Q5 What is the reason for your answer to Q4?**

**Q6 In the future, where would you prefer to be during the same procedure?**

- In the procedure room
- Outside of the procedure room
- No preference
- Unsure

**Q7 What is the reason for your answer to Q6?**

**Q8 How satisfied are you with the way the staff prepared you for the procedure?**

- Very dissatisfied
- Somewhat dissatisfied
- A little dissatisfied
- Neutral
- A little satisfied
- Somewhat satisfied
- Very satisfied

**Q9 What is the reason for your answer to Q8?**

**Q10 How could the staff have better prepared you for the procedure?**

**Q11 How satisfied are you with your choice to be inside/outside of the procedure room during the procedure?**

- Very dissatisfied
- Somewhat dissatisfied
- A little dissatisfied
- Neutral
- A little satisfied
- Somewhat satisfied
- Very satisfied

**Q12 What is the reason for your answer to Q11?**

**Q13 How important is the option to be in or outside the procedure for all procedures performed on family members?**

- Not at all important
- A little important
- Somewhat important
- Very important
- Extremely important

**Q14 What is the reason for your answer to Q13?**

**Q15 In what ways could we improve your experience of being outside the procedure room in the future?**

**Q16 Do you have any additional comments or concerns?**
